# Learning analytics of the relationships among self-regulated learning, learning behaviors, and learning performance

**DOI:** 10.1186/s41039-017-0053-9

**Published:** 2017-07-24

**Authors:** Masanori Yamada, Atsushi Shimada, Fumiya Okubo, Misato Oi, Kentaro Kojima, Hiroaki Ogata

**Affiliations:** Faculty of Arts and Science, Kyushu University, 744 Motooka, Nishiku, Fukuoka, 819-0395 Japan

**Keywords:** Learning analytics, Self-regulated learning, Learning performance

## Abstract

This research aims to investigate the relationship between self-regulated learning awareness, learning behaviors, and learning performance in ubiquitous learning environments. In order to conduct this research, psychometric data about self-regulated learning and log data, such as slide pages that learners read, marker, and annotate, was collected. The accessing activity of device types that stored the learning management system was collected and analyzed by applying path analysis and correlation analysis using data divided into high and low performers. The results indicated that the slide pages which learners read for a duration of between 240 and 299 s had positive effects on the promotion of annotation and the learning performance directly, and albeit indirectly, the enhancement of self-efficacy was affected by other self-regulated learning factors. The results of the correlation analysis indicated that self-efficacy and test anxiety are a key factor that has different effects on the number of the read slide pages in both high and low performers.

## Introduction

Learning analytics is a key subject in educational research all over the world as the findings of learning analytics studies can be applied to improve education, create learning support services, and establish learning models, among other improvements (Gray et al. 2014). One of the key issues in learning analytics is to collect learning logs using information and communication technologies (ICTs).

As ICT advances, the methods of data collection also become more various, in particular ubiquitous technologies (Yin et al. 2014). Ubiquitous technologies allow us to collect not only access logs but also location data. However, psychometric data as well as learning logs should be collected in order to analyze learners’ behaviors for the provision of effective learning support, especially learning styles such as self-regulated learning (SRL) which are thought to be helpful. SRL is closely linked to the concept of autonomy, particularly in the aspects of metacognition, motivation, and learning behavior (Schunk and Zimmerman 1998; Zimmerman 1986), which enables learners to take responsibility for their own learning.

Goda et al. (2013) suggested that psychometric data on self-regulated learning is useful regarding the prediction of the degree of help-seeking and learning performance of learners. Tseng et al. (2014) reported a significant relationship between learners’ perception of SRL, information literacy, and information-searching strategies using the Internet. Their research indicated that information literacy plays an important role in the enhancement of SRL in the ICT era. Artino Jr. and Jones II (2012) revealed that enjoyment and frustration can be positive predictors of metacognition in online learning. Other findings suggest that the use and functions of ICT promote SRL (e.g., Greene et al. 2010), whereas others have focused on developing SRL support systems (e.g., Azevedo 2005; Aleven et al. 2010). If a relationship between self-regulated learning and learning behaviors is found, the results can contribute to support learners effectively. This research aims to examine the relationship between learning behaviors, SRL awareness, and learning performance.

## Literacture review Self-regulated learning in a computer-based learning environment

When using ICT, learners can control when, what, and how they learn, without the restrictions of time, learning space, and printed materials (Cunningham and Billingsley 2003). One of the most popular platforms worldwide, the learning management system (LMS), offers the opportunity to learn outside the classroom using the Internet. To exercise control in online learning, learners have to develop self-regulated learning (SRL) skills (Yukselturk and Bulut 2007). SRL is the active learning process used to regulate and monitor learning cognition, motivation, and behavior and to set personal learning goals, including social aspects (Wolters et al. 2003; Schunk and Zimmerman 2008).

SRL is related to motivation, cognition, and self-control as it is directed toward the accomplishment of learning purposes (Pintrich 1999; Zimmerman and Paulsen 1995). SR learners are those who can prepare a learning plan, adjust it, and apply self-control and self-evaluation (Deci et al. 1996). Goda et al. (2015) suggested that high-level SR learners can control and manage their learning plan in the context of their everyday lives, using a blended learning environment.

The effects of SRL seem to be different between high and low performers. Schunk and Zimmerman (1998) further compared the learning behaviors of novice and expert SRL learners in each SRL phase (see Table 1). In the forethought phase, skillful learners could articulate their final goals as well as the necessary steps toward accomplishing the same. The features of both the goal and the steps toward it were constructive and clear. Skillful learners also tended to have internal motivation and high self-efficacy. In the performance/volitional phase, skillful learners enhanced their learning by monitoring the learning process. In the self-reflection phase, they sought to evaluate their learning performance independently and tended to attribute its quality to learning strategies and practice. The SRL features of the skillful learners in each phase support learning processes by helping teachers predict learning styles and learning performance.

**Table 1:** Differences between naïve and skillful SRLs (Schunk and Zimmerman 1998)

Classes of self-regulated learners		
Self-regulatory phases	Naïve self-regulators	Skillful self-regulators
Forethought	Nonspecific, distal goals	Specific, hierarchical goals
	Performance goal orientation	Learning goal orientation
	Low self-efficacy	High self-efficacy
	Disinterested	Intrinsically interested
Performance/volitional control	Unfocused plan	Focused on performance
	Self-handicapping strategies	Self-instruction/imagery
	Outcome self-monitoring	Process self-monitoring
Self-reflection	Avoid self-evaluation	Seeking self-evaluation
	Ability attributions	Strategy/practice attributions
	Negative self-reactions	Positive self-reactions
	Nonadaptive	Adaptive

Several scholars have also conducted studies on the computer-assisted learning environment (e.g., Azevedo 2005). Recent research has focused on SRL in an ICT-based learning environment as ICTs are now used in education and learning settings. Attitudes toward the use of ICT affect SRL. For example, Usta (2011) indicated that a negative attitude toward ICT use has a positive relationship with goal setting, time management, help seeking, and self-regulation. Greene and Azevedo (2010) indicated that learners who do well in an ICT-based environment can manage their learning using cognitive and metacognitive processes, such as ensuring the effectiveness of learning strategies, setting learning objectives, and self-monitoring. Greene et al. (2010) reviewed learning support in four types of ICT-based learning environments. The first is behaviorism, such as drill and practice, in which the same questions are asked and answered repeatedly, followed by the reception of the same feedback. The second is an adaptive or intelligent tutoring system, which supports the activation of metacognition and information retrieval. The third is hypertext and hypermedia, which allow the organization of digital learning materials using linked information. Hypertext and hypermedia work as open-learning material databases. The last one is simulation, which supports cognitive and metacognitive learning, such as information organization, hypothesizing, observation, and learning output. As such, an ICT-based learning environment supports SRL skill acquisition by indirectly promoting the use of cognitive and metacognitive learning strategies.

### Learning analytics and SRL

Learning analytics have been the subject of attention in educational research all over the world as the findings of learning analytics studies can be applied to improve education, create learning supports, establish learning models, and so on (Yin et al. 2014). One of the key issues in learning analytics is to collect learning logs using ICT. As ICT advances, the methods of data collection have been various.

Oi et al. (2015) investigated the relationship between the learning performance and the frequency of links among pages in learning materials using logs. The results revealed that high-achievement learners tended to use cognitive learning strategies, such as linking the pages and knowledge with learning materials. Goda et al. (2015) identified seven distinct learning behavior types using learning logs: procrastination, learning habit, random, diminished drive, early bird, chevron, and catch-up. They revealed that the students who had the learning habit type and the chevron type gained higher scores than the procrastination type.

One of the common issues under discussion is how psychological variables affect learning performance in a learning environment using ICT (Greene and Azevedo 2010). Winne (2010) pointed out the great possibility to enhance SRL research using ICT, but he also pointed out the importance of the psychometric data such as belief and contextual thought in SRL research. Psychometric data as well as learning logs should be collected in order to analyze learners’ behaviors for effective learning support, and in particular, learning styles such as SRL should be helpful (Roll and Winne 2015). Goda et al. (2015) focus on the access log analysis, and therefore, they did not mention the relationship between SRL and learning behaviors.

Yamada et al. (2015) indicated that self-efficacy, which is one of the factors of SRL, has a significant correlation with learning behaviors, such as highlighting and annotation. Their study indicated that SRL factors directly affect the notion of procrastination and lead to learning performance. However, their limited research did not investigate the relationships between SRL and learning behaviors. Time-related learning awareness, which plays an important role in SRL awareness, can affect learning awareness and performance, such as time-management awareness and skill (Zimmerman 1990; Eilam and Aharon 2003; Bernard et al. 2009; Kizilcec et al. 2016). Eilam and Aharon (2003) investigated the process and effects of students’ learning plans and highlighted the differences in the learning planning process between high and low performers. Yamada et al. (2016) demonstrated the causal relationship between time-management awareness, the submission time for learning outcomes on the LMS, and learning performance. Time-related learning awareness and behaviors seem to impact learning awareness and behaviors according to previous research; however, these previous studies did not examine the effects of time-related learning behaviors using learning logs, and the relationships between learning behaviors, SRL awareness, and learning performance. In particular, these previous studies did not consider the perspective of ordinary learning behaviors such as the time used for reading per page of the learning material. This study considered time-related learning behaviors, in particular the reading time for each page of the learning material, and then calculated the number of pages for the reading time segmented by per minute. As a result, using learning logs on the learning support system, this study investigated the effects of reading time per page of the learning material on the use of learning strategies, SRL awareness, and learning performance. In particular, these previous studies did not consider the perspective of ordinary learning behaviors such as the time used for reading per page of the learning material. This study considered time-related learning behaviors, in particular the reading time for each page of the learning material, and then calculated the number of pages for the reading time segmented by per minute. As a result, using learning logs on the learning support system, this study investigated the effects of reading time per page of the learning material on the use of learning strategies, SRL awareness, and learning performance.

If a relationship between self-regulated learning and learning behaviors is found, the results may be used to support learners effectively. Therefore, this exploratory research aims to investigate the relationships between the number of learning material slides in every reading time, the use of annotations and marker functions, SRL awareness, and learning performance.

## Methods

### Participants and class

This research was conducted on two information technology courses. One was a 15-week course (course 1), and the other was an 8-week course (course 2). The participants were 127 freshman university students in information technology classes (93 students for course 1 (Engineering 57, Science 13, Medical 2, Literature 2, Economics 1, Education 4, and Art technology, 1) and 34 students for course 2 (Engineering 2, Pharmacy 8, Education 7, Medical 9, Law 5, Literature 2, Dentistry 1)). These classes were semi-obligatory for pre-service students who desired to obtain a teaching qualification. All the students had fundamental computer skills, such as Microsoft office, email, and Net surfing.

**Fig. 1:**
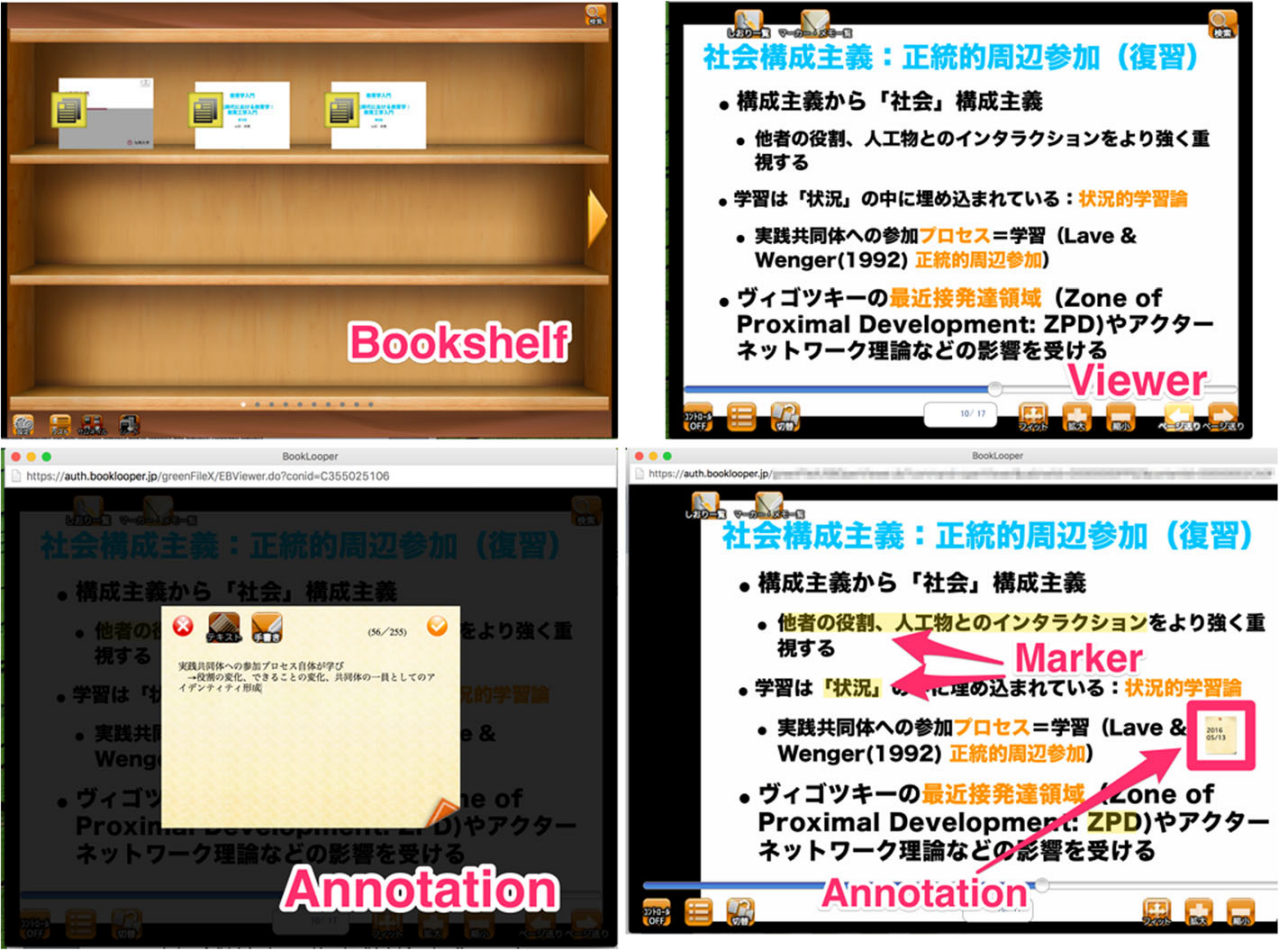
The interface of DLMR “BookLooper”

The time allotted for one class per week was 90 min. Teachers asked the students to bring their laptop to the classes. In the first week, the teachers explained the usage and functions of a digital learning material reader (DLMR). The teachers distributed digital learning materials to the students with the use of DLMR and encouraged the students to read the materials in advance before every class. A DLMR allowed the students to access the learning materials on devices such as laptops and smartphones and to use marking and annotation functions whenever and wherever the Internet was available. Figure 1 shows the interface of the DLMR. The DLMR allows the learners to promote the use of cognitive learning strategies such as marker and annotation (Chen et al. 2012). This system has zoom and word retrieve functions, but we did not ask the learners to use these functions, including marker and annotation. The students were required to respond to questionnaires before starting the instruction in the first class (pre-class questionnaire). In every class, learners took the comprehension test at the beginning. After the comprehension multiple-choice test (10 questions about the current content) in every class, the students took the lecture and worked on basic computer science contents, such as encryption technology, image processing, ontology, and programming. At the end of the last class, the teachers required the learners to respond to the same questionnaire as the pre-questionnaire (post-class questionnaire).

### Data collection and analysis

Two methods were used for the data collection: a questionnaire and log. The Motivated Strategies and Learning Questionnaire (MSLQ: Pintrich and DeGroot 1990), which consists of five factors (Self-Efficacy (SE), Intrinsic Value (IV), Cognitive Strategies (CS), Self-Regulation (SR), Test Anxiety (TA); 44 items in sum, rated on a seven-point Likert scale; see the Appendix) was used for the subjective evaluation of learners’ SRL awareness. The students were asked to complete the MSLQ both before and after the classes. The differences between their responses on the pre-class and post-class questionnaires were analyzed. The second method of data collection was a log that recorded the number of pages that the learners had read and their behavior of marking, bookmarking, and annotating. The number of reading pages was counted according to the following reading duration in every 1 min: 10–59, 60–119, 120–179, 180–239, 240–299, 300–359, 360–419, 420–479, 480–539, 540–599, and over 600 s. However, the slides for which the duration was from 1 to 10 s were eliminated from the data, because the learners did not read the contents (just skipped). The reading time was calculated from the difference between and the sum of the time of page flip logs (forward and back). We counted the frequency of marking and annotation in total throughout the course. Bookmark log was not used for this research, due to very few logs (49 out of 245,096 logs). The learning performance is the final score.

## Results

### Descriptive data and *t* test for SRL

The dataset, retrieved from the descriptive data and the results of the test for MSLQ factors, consisted of 121 learners’ items after eliminating the missing data. MSLQ data were analyzed using the *t* test in order to evaluate the differences between pre- and post-course responses from the viewpoint of the improvement of SRL. Path analysis was employed to investigate the relationships between SRL, learning behaviors, and learning performance. Table 2 shows the descriptive data (averages, standard deviations) of MSLQ factors and the results of the *t* test, and Tables 3 and 4 show the descriptive data of the pages, which learners read, and the frequency of marker and annotation function use and the final score.

**Table 2:** The descriptive data and the results of the *t* test for MSLQ

Item (minimum-maximum)	Average (SD) before the course	Average (SD) after the course	*t*	Sig
Self-efficacy (9 questions) (9–63)	32.54 (8.96)	36.42 (9.20)	*t*(120) = 4.51	*p* < 0.001
Intrinsic value (9 questions) (9–63)	45.21 (6.29)	44.31 (8.15)	*t*(120) = 1.20	
Cognitive strategies (13 questions) (13–91)	60.12 (7.79)	60.63 (8.69)	*t*(120) = 0.68	
Self-regulation (9 questions) (9–63)	36.26 (5.63)	36.49 (6.22)	*t*(120) = 0.42	
Test anxiety (4 questions) (4–28)	15.17 (4.45)	16.07 (4.34)	*t*(120) = 2.21	*p* < 0.05

Note: The min-max scores of all the domains in the questionnaire were calculated based on the Likert scale of 1–7

**Table 3:** The descriptive data of the pages that learners read

	Average (SD)	Minimum	Maximum
All pages (over 1 s)	1492.63 (1259.09)	0	5365
10–59 s	169.32 (140.66)	0	692
60–119 s	35.01 (29.67)	0	135
120–179 s	15.72 (13.80)	0	74
180–239 s	8.31 (7.75)	0	44
240–299 s	5.59 (5.57)	0	27
300–359 s	3.83 (4.35)	0	30
360–419 s	2.49 (2.72)	0	19
420–479 s	1.80 (1.86)	0	9
480–539 s	1.54 (1.73)	0	8
540–599 s	1.21 (1.74)	0	11
600 s	6.30 (6.98)	0	47

**Table 4:** The descriptive data of the marker and annotation functions use and final score

	Average (SD)	Minimum	Maximum
Marker use	5.99 (12.15)	0	83
Annotation use	4.23 (9.72)	0	62
Final score	85.84 (11.72)	39.93	100.00

The results of the *t* test showed that self-efficacy and test anxiety were higher in the post-class responses compared to the pre-class responses; that is, learning behaviors seemed to improve learners’ self-efficacy in the class (*p* < 0.001, *t*(120) = 4.51, effect size (*d*) = 0.5843) but promote test anxiety (*t*(129) = 2.21, *p* < 0.05, effect size (*d*) = 0.4754). Significant differences between the pre- and post-questionnaire results were not found for other factors. One interesting point is that there was no significant difference between the pre- and post-stage in intrinsic value, but the SD increased even though the average decreased. In this class, the individual differences in the perception of intrinsic value appeared to be significant.

As for the descriptive data on reading pages in all and each duration displayed in Table 3, many learners in these classes read the slides for 10 to 299 s, because the average number of pages did not exceed the standard deviation. However, with the data on the number of pages that learners read for over 300 s, the standard deviation is higher than the average page number. This means that the individual differences in the number of pages that learners read for over 300 s increased. Some learners took a much longer time to read the page, while others did not.

### Path analysis

**Fig. 2:**
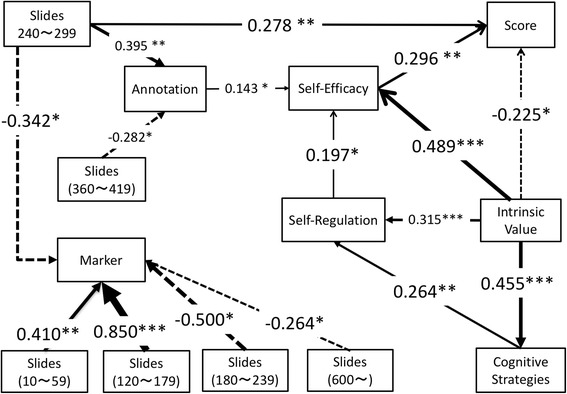
The results of path analysis

It is important to consider the kinds of learning behavior that improved learners’ SRL factors and learning performance. In order to investigate the overall relationships, a path analysis was employed. Each variable of the SRL is the average difference between the post- and pre-MSLQ questionnaires (see Fig. 2 for the results). The indicators of the model fitting are acceptable: CFI 0.963, TLI 0.948, RMSEA = 0.036, *χ* ^2^(41) 47.401, *p* = 0.228. The results indicated that the numbers of slides that learners read from 10 to 59 s and 120 to 179 s promoted the use of the marker function more. However, the numbers of slides read from 180 to 239 s, 240 to 299 s, and over 600 s had a negative effect on the use of the marker function, implying a significant difference between the two segments that had positive effect. One segment of the “slide number” that learners read from 240 to 299 s promoted the use of the annotation function; however, the slide numbers that learners read from 360 to 419 s had a negative effect on its use. The results also revealed the mediated functions of SRL perception between learning behaviors and learning performance. The number of slides that the learners read from 240 to 299 s had both direct and indirect positive effects on learning performance. This time range from 240 to 299 s also promoted the use of annotations. The use of annotations promoted the perception of self-efficacy, and in turn, self-efficacy enhanced learning performance. The awareness of intrinsic value is a fundamental perception that affects other SRL factors and indirectly affects learning performance. The awareness of intrinsic value enhanced the sense of awareness of cognitive learning strategies, self-regulation, and self-efficacy. The awareness of cognitive strategies use enhanced self-regulation, and consequently, self-regulation enhanced self-efficacy. Interestingly, internal value has a directly negative effect on the enhancement of learning performance; that is, learners who placed importance on the learning contents in this class significantly gained a lower test score than those who did not regard this class as important for them. However, intrinsic value indirectly had positive effects on learning performance, mediated by self-efficacy. We did not find any relationship with test anxiety.

### Correlation analysis

Regarding SRL effects on learning performance, in this phase, a correlation analysis (Spearman’s rho) was conducted in order to investigate the relationship between SRL and learning performance, divided into two groups: high performance and low performance. The purpose of the analysis was to understand the path analysis results as previous research indicated the differences in SRL awareness between high and low performers (e.g., Zimmerman 1990; Schunk and Zimmerman 1998; Nandagopal and Ericsson 2012).

The learners who gained a score higher than the average of plus 1 SD were categorized into the high performance group (*N* = 14), and those who gained a score less than the average score of minus 1 SD were allocated to the low performance group (*N* = 19). Tables 5 and 6 show the results of the correlation analysis. The results show the differences between high and low performers. With regard to self-efficacy, the results showed no correlation with test anxiety with the high performer data; however, a high correlation was found with the low performer data. Concerning the correlation between self-efficacy and the slide page number in each reading time segment (1-min segment), almost all the correlations were weak negative; however, very weak to weak positive correlations were found in the low performer data. With regard to intrinsic value, very weak to weak positive correlations with the slide page number in each segment were confirmed; however, a weak negative correlation was found in the low performer data. About the awareness of cognitive learning strategy use, there were weak positive correlations with cognitive strategy use, but no correlation was found with the low performer data. Concerning test anxiety, negative correlations with the slide page number in each segment less than 360 s were confirmed, but mainly a weak positive correlation was found in the low performer data. On the other hand, for the time duration over 360 s, weak positive correlations between test anxiety and the number of pages that learners read were confirmed. With regard to the relationships between cognitive learning strategy use and the number of pages, there were significant differences between high and low performers. The correlation results with the high performer data confirmed the negative correlation between the number of pages and marker use and between the number of pages and annotation use. On the other hand, middle to strong positive correlations between the number of pages and marker use were confirmed. There were very slight negative correlations between the number of pages and annotation use, except for the number of pages with the reading time from 480 to 539 s in the low performer group.

**Table 5:** The results of the correlation analysis (Spearman’s rho) for high performer data (*N* = 14)

	1	2	3	4	5	6-1	6-2	6-3	6-4	6-5	6-6	6-7	6-8	6-9	6-10	6-11	6-12	7	8
1: Self-efficacy	1.000																		
2: Intrinsic value	0.732	1.000																	
3: Cognitive learning strategies	0.555	0.720	1.000																
4: Self-regulation	0.518	0.642	0.705	1.000															
5: Test anxiety	0.090	0.285	−0.047	−0.054	1.000														
6: Slide																			
6-1: All	−0.197	0.183	−0.101	−0.090	−0.053	1.000													
6-2: 10–59 s	−0.117	0.152	−0.223	−0.106	−0.007	0.934	1.000												
6-3: 60–119 s	0.075	0.322	−0.046	−0.138	−0.055	0.899	0.868	1.000											
6-4: 120–179 s	−0.077	0.095	−0.207	−0.340	−0.134	0.786	0.731	0.856	1.000										
6-5: 180–239 s	−0.196	0.024	−0.197	−0.137	0.275	0.865	0.808	0.823	0.880	1.000									
6-6: 240–299 s	−0.116	0.230	0.025	−0.174	−0.102	0.857	0.722	0.874	0.907	0.825	1.000								
6-7: 300–359 s	−0.098	−0.117	−0.452	−0.433	−0.198	0.563	0.676	0.744	0.777	0.677	0.586	1.000							
6-8: 360–419 s	−0.178	0.207	−0.136	−0.185	0.355	0.705	0.723	0.719	0.449	0.474	0.569	0.471	1.000						
6-9: 420–479 s	−0.470	0.025	−0.222	−0.085	0.270	0.759	0.727	0.606	0.457	0.520	0.578	0.440	0.761	1.000					
6-10: 480–539 s	−0.277	0.154	0.034	−0.112	0.192	0.745	0.597	0.561	0.450	0.446	0.635	0.101	0.603	0.687	1.000				
6-11: 540–599 s	−0.151	0.117	−0.157	−0.306	−0.001	0.598	0.453	0.742	0.652	0.601	0.745	0.543	0.606	0.745	0.472	1.000			
6-12: Over 600 s	−0.300	0.088	−0.208	−0.165	0.228	0.669	0.709	0.627	0.567	0.477	0.574	0.622	0.704	0.883	0.584	0.384	1.000		
7: Marker	0.114	0.177	0.232	0.403	0.060	−0.124	−0.029	−0.232	−0.250	0.011	−0.335	−0.222	−0.222	−0.169	−0.389	−0.483	−0.261	1.000	
8: Annotation	0.341	0.473	0.636	0.300	0.152	−0.352	−0.427	−0.318	−0.423	−0.468	−0.320	−0.554	−0.312	−0.377	0.029	−0.233	−0.374	0.334	1.000

**Table 6:** The results of the correlation analysis (Spearman’s rho) for high performer data (*N* = 19)

	1	2	3	4	5	6-1	6-2	6-3	6-4	6-5	6-6	6-7	6-8	6-9	6-10	6-11	6-12	7	8
1: Self-efficacy	1.000																		
2: Intrinsic value	0.415	1.000																	
3: Cognitive learning strategies	0.551	0.536	1.000																
4: Self-regulation	0.598	0.424	0.559	1.000															
5: Test anxiety	0.647	0.076	0.350	0.711	1.000														
6: Slide																			
6-1: All	0.066	−0.230	−0.185	−0.150	0.037	1.000													
6-2: 10–59 s	0.163	−0.204	−0.115	−0.034	0.145	0.954	1.000												
6-3: 60–119 s	0.070	−0.339	−0.182	−0.113	0.128	0.941	0.955	1.000											
6-4: 120–179 s	0.320	−0.150	0.043	0.085	0.320	0.867	0.909	0.899	1.000										
6-5: 180–239 s	0.096	−0.119	−0.023	0.008	0.152	0.875	0.899	00.908	0.965	1.000									
6-6: 240–299 s	0.075	−0.192	0.000	0.013	0.007	0.827	0.839	0.890	0.851	0.929	1.000								
6-7: 300–359 s	0.234	−0.253	0.074	−0.073	0.131	0.766	0.797	0.845	0.834	0.889	0.854	1.000							
6-8: 360–419 s	0.201	−0.181	−0.030	−0.059	0.191	0.841	0.848	0.895	0.885	0.896	0.799	0.895	1.000						
6-9: 420–479 s	0.088	−0.340	−0.017	−0.010	−0.153	0.679	0.732	0.738	0.608	0.660	0.754	0.740	0.642	1.000					
6-10: 480–539 s	0.008	−0.280	−0.117	−0.185	0.046	0.724	0.711	0.763	0.679	0.741	0.779	0.734	0.551	0.485	1.000				
6-11: 540–599 s	0.031	−0.277	0.047	0.070	0.230	0.592	0.552	0.649	0.568	0.621	0.623	0.739	0.739	0.503	0.442	1.000			
6-12: Over 600 s	0.094	−0.257	−0.155	−0.049	0.238	0.900	0.898	0.950	0.880	0.854	0.833	0.744	0.824	0.547	0.784	0.575	1.000		
7: Marker	0.189	−0.157	−0.058	0.204	0.081	0.738	0.713	0.730	0.723	0.675	0.572	0.526	0.650	0.418	0.557	0.130	0.751	1.000	
8: Annotation	0.328	0.266	−0.010	0.198	0.316	0.028	0.023	0.016	0.038	−0.068	−0.193	−0.181	0.133	−0.207	−0.352	−0.034	0.062	0.201	1.000

## Discussion

This research aims to investigate the relationships between learning behaviors, SRL awareness, and learning performance. We found mainly positive relationships between them. The number of slides that the learners read from 120 to 179 s and from 10 to 59 s promoted the frequent use of the marker function; however, the numbers of pages that learners read from 180 to 239 s, from 240 to 299 s, and over 600 s inhibited the use of the marker function. In order to consider this point in detail, we referred to the results of the correlation analysis. The results revealed a positive correlation between the number of pages and marker use, and both weak negative and positive correlations between marker use and SRL in low performer data.

The number of slides read from 240 to 299 s had both direct and indirect positive effects on the enhancement of the final score. In order to consider this linkage, considering the indirect path of the number of slides that learners read from 240 to 299 s, the results showed that the learners tend to add annotations on the slides from 240 to 299 s. One possible reason for the relationship between learning behaviors and SRL is that the learning behaviors of the learners depend on the time duration. Reading comprehension requires learners to complexly process input information, such as letter and word recognition, and knowledge integration (Van Gelderen et al. 2007). Walczyk et al. (2007) suggested the relationship between the learning process and disruptive compensatory reading strategies, in that deep reading processes such as semantic encoding seem to require more time for information processing. The time duration from 240 to 299 s seems to be the threshold to engage with the learning contents, according to the results of the path analysis. It indicated that learners attempted to comprehend the learning contents and pointed to both clear and unclear parts in the slide that they read for this duration. Therefore, annotation enhanced self-efficacy, which is one of the important elements of SRL. These results were supported by Bernacki et al. (2012), who suggested the effectiveness of annotation in the enhancement of SRL. However, marker use did not promote any SRL elements collected in this research, which differs from the results suggested by Bernacki et al. (2012). A possible reason could be the correlations between SRL and its functions. In the high performer data, weak- to middle-level positive correlations between SRL and its function use were confirmed; in particular, annotation had a strong positive correlation with SRL. From these results, the effects of marker use on SRL can be restrained by annotation use.

Intrinsic value is the key SRL element in the research results. Intrinsic value enhances self-efficacy, self-regulation, and the awareness of cognitive learning strategy use. In addition, it has indirect effects on learning performance, mediated by self-efficacy. Intrinsic value seems to be related with the relevance to learners’ situation, which enhances learning motivation (Keller 2009). In the case of learners being aware of self-efficacy, self-regulation, and cognitive learning strategies, learners seemed to gain a high score. However, one point that differed from previous research is that there is a direct negative relationship between intrinsic value and learning performance in the path analysis results. In order to investigate this point, we conducted Spearman’s correlation analysis, using high and low performers’ data. The results of the correlation analysis indicated very weak to weak positive correlation between intrinsic value and the number of pages in each segment, except for the duration from 300 to 359 s; however, there was a small negative correlation between the intrinsic value of this class and the number of slides read. One possible reason for this is the indirect effects of test anxiety. With the higher performer data, the intrinsic value had a positive relationship between other SRL factors. High performers seemed to control their SRL skill well including intrinsic value and succeeded in gaining high scores, as suggested by Schunk and Zimmerman (1998). The low performer data also indicated a middle- and high-level correlation between SRL factors, but the difference between high and low performers arose from test anxiety. With the higher performer data, we found a low level of correlation between the internal value and test anxiety (0.285), but with the low performer data, there was almost no correlation (0.076). With the low performer data, however, test anxiety had a middle-high-level positive correlation with self-efficacy (0.647), but the high performer data did not (0.090) indicate similar results. Low performers seemed to recognize test anxiety as an individual factor, but high performers seemed to focus on being aware of intrinsic value with the feeling of test anxiety. These results seem to be consistent with Schunk and Zimmerman (1998), who indicated that an expert self-regulated learner tends to focus on self-evaluation through self-reflection.

Cognitive learning strategy use is one of the fundamental SRL elements for the quality learning outcomes (e.g., Pintrich and DeGroot 1990). The use of markers did not affect the learning performance and SRL directly, but annotation affected self-efficacy and indirectly affected learning performance in this research. A possible reason is that the annotation feature requires learners to act more cognitively on learning material, but the marker seems to do more cognitively. Annotation, which supports cognitive learning, is one of the most effective tools and learning strategies for the enhancement of motivation and learning outcomes on hypermedia. Chen and Huang (2014) suggested that annotation supports learners’ attention in learning and SRL and, as a result, enhances learning outcomes. Shang (2016) revealed that online annotation on the learning materials enhances the motivation and learning material comprehension significantly. In contrast, the marker is one of the most common tools used with e-books, but the use of this function did not have any significant effects on the enhancement of learning outcomes (van Horne et al. 2016). van Horne et al. (2016) also suggested that the use of highlighting was associated with the delay in reading time and prior use of paper-based textbooks, which are concerned with re-reading behavior. In fact, correlation analysis revealed that positive middle-level correlations were found between awareness and behaviors of the cognitive learning strategies in high learning performers, but not in low learning performers. Annotation seems to be a “cost-effective” tool for learners to enhance their learning outcome, compared with the marker tool. When learners use the marker function and look back at the highlighted pages, learners seem to consider the meaning of the marker and associated information with the marker. Annotation simply supports the learner’s understanding of learning materials. This feature seems to promote the enhancement of self-efficacy and, indirectly, the learning performance.

However, this correlation analysis is very limited, using Spearman’s rho for only 33 datasets. Therefore, regarding further research, we need to investigate the effects of internal value and test anxiety to increase the dataset. One point that we should consider is that the average score of these classes was very high, 85 out of 100. This means that it seems to be very difficult to find a significant relationship with learning performance. These points should be considered for further research.

## Conclusion

This research investigated the relationship between SRL factors, learning behaviors, and learning performance. The results showed that the number of slides read from 240 to 299 s indirectly had a positive influence on the enhancement of SRL learning performance. Annotation, which is the learning behavior related with learning strategy, mediated between the slides and SRL. Among the SRL factors, all the relationships between them were positive, but the internal value had a negative effect on the final score. In order to investigate this point in detail, we investigated the differences between high and low performers using correlation analysis. The results showed different correlations between intrinsic value and the number of slides read, with positive correlations among high performers and negative correlations among low performers.

This research contributes useful suggestions to the learning analytics research field. SRL is an important educational theory and concept in education, globally. Previous research has tended to capture learners’ SRL awareness using questionnaires or observations (e.g., Pintrich and DeGroot 1990; Zimmerman 2008; Cho and Jonassen 2009; Jansen, van Leeuwen, Janssen, Kester, and Kalz 2016). This research examined learning behaviors related with SRL using learning logs and provided viewpoints to consider SRL awareness, which is different from previous studies in SRL research. Learning analytics research tends to focus on the use of logs by utilizing ICT. However, learning analytics research mixed with educational psychology methods, such as those used here, can suggest the background of learning behaviors (learning logs). Thus, learning analytics blended with educational psychology methods can provide researchers and practitioners with various viewpoints on educational evaluation.

Additionally, this research makes a practical contribution toward designing effective instruction. This research recommends that teachers design their instruction for the enhancement of learners’ self-efficacy by using the awareness of cognitive learning strategies that are directly effective in enhancing internal value and indirectly effective in enhancing self-regulation. For instance, the introduction of useful cognitive learning strategies in a teacher’s class may be a simple idea; however, such introduction also entails the use of an effective instructional design method. Further, this research identified several problems on which researchers can focus as part of future research. Particularly, future research can address the following three issues.

Firstly, the effects of mobile usage should be investigated to promote the use of learning with mobiles. In this study, only 4 out of 90 learners used mobiles in order to read the slides. Mobile usage can enable and encourage learners to learn anywhere and at any time. The mobility seems to enhance SRL awareness. Sha et al. (2012) pointed out learners’ academic achievement and motivation effect relating to the use of a mobile-based learning environment from the viewpoint of SRL. We did not find the significant effects of the mobile device use, due to the small mobile users; therefore, we should consider their suggestion for further research. Secondly, the duration of accessing days should be added as a variable for the prediction of learning performance. Regular access can influence the SRL awareness and can lead to an improvement in learning performance. Lastly, data analysis can include the addition of data from other classes. This research used data from two classes, but we should include data from other classes in order to extract a useful and versatile model for teaching and learning support.

## Appendix

**Table 7:** Motivated strategies and learning questionnaire (Pintrich and DeGroot 1990)

Factor	Item
Self-efficacy	Compared with other students in this class, I expect to do well.
	I’m certain I can understand the ideas taught in this course.
	I expect to do very well in this class.
	Compared with others in this class, I think I’m a good student.
	I am sure I can do an excellent job on the problems and tasks assigned for this class.
	I think I will receive a good grade in this class.
	My study skills are excellent compared with those of other students in this class.
	Compared with other students in this class, I think I know a great deal about the subject.
	I know that I will be able to learn the material for this class.
Intrinsic value	I prefer class work that is challenging so I can learn new things.
	It is important for me to learn what is being taught in this class.
	I like what I am learning in this class.
	I think I will be able to use what I learn in this class in other classes.
	I often choose paper topics I will learn something from even if they require more work.
	Even when I do poorly on a test, I try to learn from my mistakes.
	I think that what I am learning in this class is useful for me to know.
	I think that what we are learning in this class is interesting.
	Understanding this subject is important to me.
Test anxiety	I am so nervous during a test that I cannot remember facts I have learned.
	I have an uneasy, upset feeling when I take a test.
	I worry a great deal about tests.
	When I take a test, I think about how poorly I am doing.
Cognitive strategy use	When I study for a test, I try to put together the information from class and from the book.
	When I do homework, I try to remember what the teacher said in class so I can answer the questions correctly.
	It is hard for me to decide what the main ideas are in what I read. (R)
	When I study, I put important ideas into my own words.
	I always try to understand what the teacher is saying even if it doesn’t make sense.
	When I study for a test, I try to remember as many facts as I can.
	When studying, I copy my notes to help me remember material.
	When I study for a test, I practice saying the important facts over and over to myself.
	I use what I have learned from old homework assignments and the textbook to do new assignments.
	When I am studying a topic, I try to make everything fit together.
	When I read material for this class, I say the words over and over to myself to help me remember.
	I outline the chapters in my book to help me study.
	When reading, I try to connect the things I am reading about with what I already know.
Self-regulation	I ask myself questions to make sure I know the material I have been studying.
	When work is hard, I either give up or study only the easy parts. (R)
	I work on practice exercises and answer end of chapter questions even when I don’t have to.
	Even when the study materials are dull and uninteresting, I keep working until I finish.
	Before I begin studying, I think about the things I will need to do to learn.
	I often find that I have been reading for class but don’t know what it is all about. (R)
	I find that when the teacher is talking, I think of other things and don’t really listen to what is being said. (R)
	When I’m reading, I stop once in a while and go over what I have read.
	I work hard to get a good grade even when I don’t like a class.

*R* reflected item
